# A continuous kinetic assay for protein and DNA methyltransferase enzymatic activities

**DOI:** 10.1186/s13072-015-0048-y

**Published:** 2015-12-15

**Authors:** Shai Duchin, Zlata Vershinin, Dan Levy, Amir Aharoni

**Affiliations:** Departments of Life Sciences, Ben-Gurion University of the Negev, 84105 Be’er Sheva, Israel; The Shraga Segal Department of Microbiology, Immunology and Genetics, Ben-Gurion University of the Negev, 84105 Be’er Sheva, Israel; The National Institute for Biotechnology in the Negev (NIBN), Ben-Gurion University of the Negev, 84105 Be’er Sheva, Israel

## Abstract

**Background:**

Methyltransferases (MTs) catalyze the *S*-adenosylmethionine (SAM)-dependent methylation of a wide variety of protein and DNA substrates. Methylation of lysine, arginine or cytosine regulates a variety of biological processes including transcriptional activation and gene silencing. Despite extensive studies of the cellular roles of MTs, their quantitative kinetic characterization remains challenging. In the past decade, several assays have been developed to monitor methyl transfer activity utilizing different approaches including radiolabeling, antibodies or mass-spectrometry analysis. However, each approach suffers from different limitation and no easy continuous assay for detection of MT activity exists.

**Results:**

We have developed a continuous coupled assay for the general detection of MTs activity. In this assay, the formation of *S*-adenosylhomocysteine (SAH) product is coupled NAD(P)H oxidation through three enzyme reactions including glutamate dehydrogenase leading to absorbance changes at 340 nm. The utility and versatility of this assay is demonstrated for SET7/9 and SETD6 with peptides and full length protein substrates and for *M.Hae*III with a DNA substrate.

**Conclusions:**

This study shows a simple and robust assay for the continuous monitoring of MT enzymatic activity. This assay can be used for the determination of steady-state kinetic enzymatic parameters (e.g., *k*_cat_ and *K*_M_) for a wide variety of MTs and can be easily adapted for high-throughput detection of MT activity for various applications.

**Electronic supplementary material:**

The online version of this article (doi:10.1186/s13072-015-0048-y) contains supplementary material, which is available to authorized users.

## Background

Methylation is a common covalent modification of nucleic acids and proteins. Recently, methylation has emerged as a prominent posttranslational modification of proteins regulating diverse cellular signaling pathways that influence cell survival, growth, and proliferation. Disruption of methylation is thought to fundamentally impact the initiation and progression of many biological processes, leading to altered cellular phenotypes and the development of diseases [1–4]. Methylation of lysine residues in target proteins is performed by protein lysine (K) methyltransferases (PKMTs) [5, 6]. Currently, there are over 60 candidates and known members of this enzyme family, the vast majority of which contain a conserved SET domain that is responsible for the enzymatic activity [5, 6]. Methylation also takes place on genomic DNA on cytosine bases in CpG dinucleotide repeats [7]. These regions of the genome are enriched in transcriptionally repressed chromatin and methylation mediates epigenetic silencing within these domains [8]. Thus, DNA methylation was shown to play a crucial role in the epigenetic control of gene expression [9].

All methyltransferases (MTs) utilize the *S*-adenosylmethionine (SAM) as a universal methyl donor leading to the generation of *S*-adenosylhomocysteine (SAH) following methylation. Due to the central biological roles of MTs, it is important to biochemically characterize and quantitatively measure their catalytic activity. Previous efforts for the development of quantitative assays for MTs activity rely on the detection of methylated product or the formation of SAH [10, 11]. Radioactive assays utilizing ^3^H-SAM are considered the most sensitive and reliable but require chromatographic separation and thus tend to be slow and labor intensive [12]. Recently, more advanced and rapid radioactive assays were developed to allow the detection of many MTs reactions in parallel [13–16]. More recently, fluorescent [17], antibody-based immunoassays or reading domain-based assays [18–20] for the detection of methylated lysine, arginine and cytosine were developed utilizing enzyme-linked immunosorbent assay (ELISA) or fluorescence resonance energy transfer (FRET) [21–23]. These assays based on fluorescence or absorbance are highly sensitive but are not general for the detection of a wide range of MT activities because a specific antibody for each substrate and/or type of modification must be used.

Assays for the detection of SAH [24–26] have the advantage of providing a general detection method for MTs regardless of the protein or DNA acceptor. Previously, the accumulation of SAH product at high concentration was shown to inhibit most MTs activity [27, 28]. The coupled assay can overcome this problem by preventing the accumulation of the SAH following MTs activity. Several coupled enzyme assays have been developed for SAH detection including the conversion of SAH to adenosine and homocysteine enabling the colorimetric or fluorescent detection with thiol reactive chromophores or fluorophores, respectively [26, 28, 29]. However, these assays were not utilized in a continuous manner due to possible interference from protein cysteine residues and the presence of thiol reducing reagents. An alternative approach utilizes SAH nucleosidase (SAHN) [30] to generate adenine and *S*-ribosylhomocysteine followed by adenine deaminase (ADE) to generate hypoxanthine [24]. This reaction can be followed continuously at 265 nm; however, due to the high absorbance of proteins at 280 nm, this approach is difficult to apply for MTs detection. An extension of this assay that includes xanthine oxidase for the generation of fluorogenic xanthine derivatives was commercialized but still suffers from limited dynamic range.

**Fig. 1:**
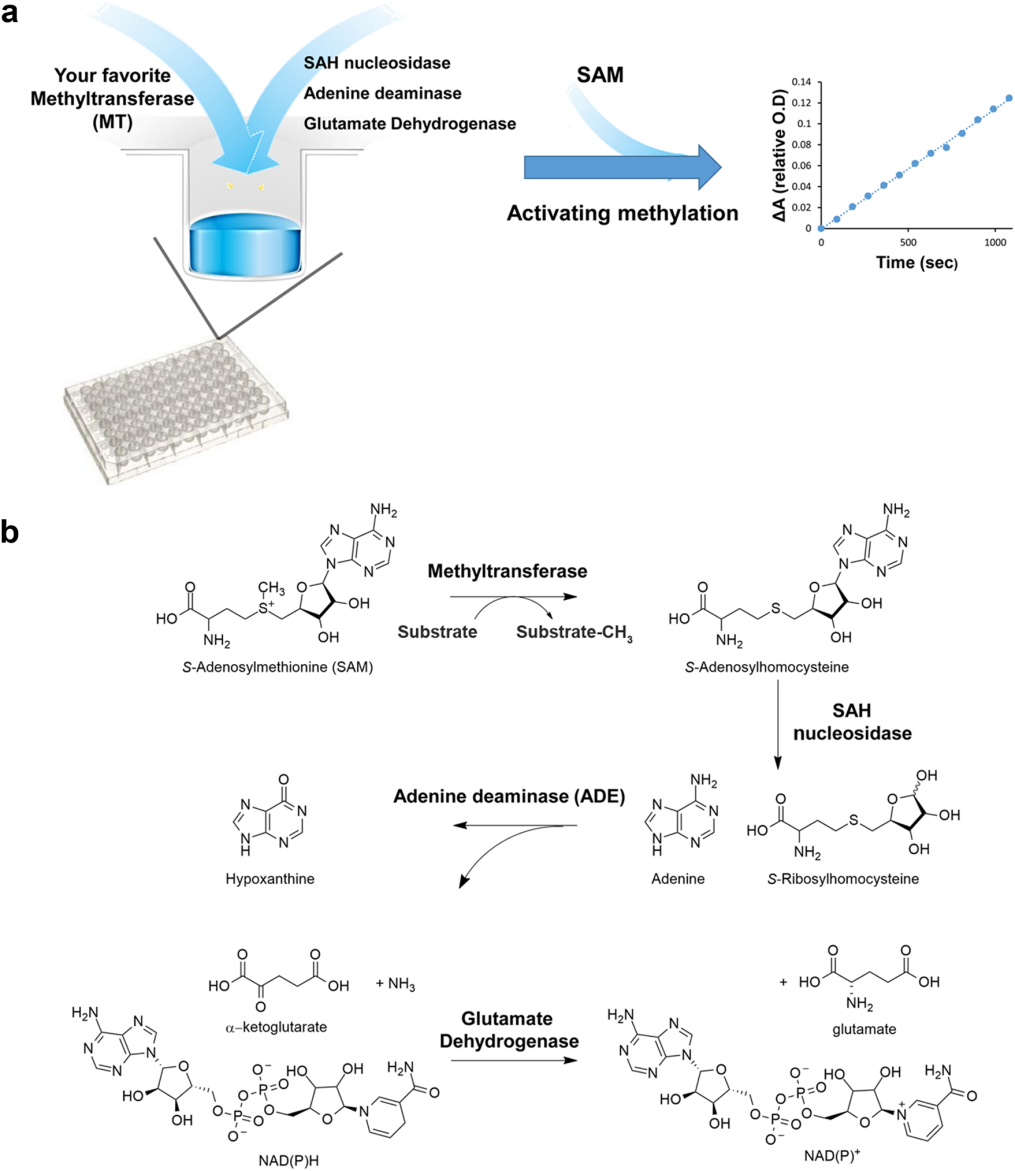
General diagram for the continuous coupled assay for MTs activity. **a** In this assay, MTs activity is coupled to three enzymes: SAH nucleosidase (SAHN), adenine deaminase (ADE) and glutamate dehydrogenase to couple methyl transfer activity to NADPH oxidation. As a result, methyl transfer activity is directly monitored by a decrease in absorbance at 340 nm. **b** *Diagram* describing the chemical structure of SAM, SAH and all other products derived from the activity of the three coupled enzyme

Here, we report the establishment of a coupled continuous assay for MTs that is based on monitoring SAH formation during the MT reaction. We have utilized SAHN, ADE and glutamate dehydrogenase [31] to couple enzymatic MT activity to NADPH oxidation (Fig. 1). NADPH oxidation is then followed at 340 nm allowing a convenient and robust detection of MT activity using standard cuvettes or multi-well plate format. Since methylation is the rate-limiting step in the coupled assay (see below), the rate of NADPH oxidation reflects the rate of methylation. We have shown that this assay is highly versatile allowing the quantitative detection of MTs activity toward peptide or protein substrates and for the detection of DNA MTs activity. This coupled assay permits the Michaelis–Menten (MM) analysis of MTs catalytic activity for mechanistic studies and is readily adaptable for high-throughput screening for the discovery of novel MTs inhibitors.

## Results

### Development of a coupled continuous assay for MTs

To establish a continuous assay for MTs activity that monitors SAH formation, the transfer of a methyl group to peptide, protein or DNA must be coupled to additional reactions that lead to measurable change in absorbance/fluorescence. Previous work has utilized SAHN and ADE for the conversion of SAH to hypoxanthine and ammonia [24]. These two coupled reactions were shown to be non-rate limiting enabling the efficient coupling of methyl transfer reaction to hypoxanthine formation that is monitored at 265 nm [24]. However, measuring MTs activity at 265 nm is highly problematic due to the high absorbance of proteins at 280 nm and the inability to utilize standard cuvettes or multi-well plates due to their high absorbance at this wavelength. To overcome these limitations, we have coupled the activity of SAHN and ADE with glutamate dehydrogenase that utilizes ammonia and α-ketoglutarate to generate glutamate while oxidizing NADPH. The coupling of this reaction to MTs activity allows monitoring the continuous change in absorbance at 340 nm due to NADPH oxidation (Fig. 1) which linearly correlates with the reduction of the SAM concentration.

**Fig. 2:**
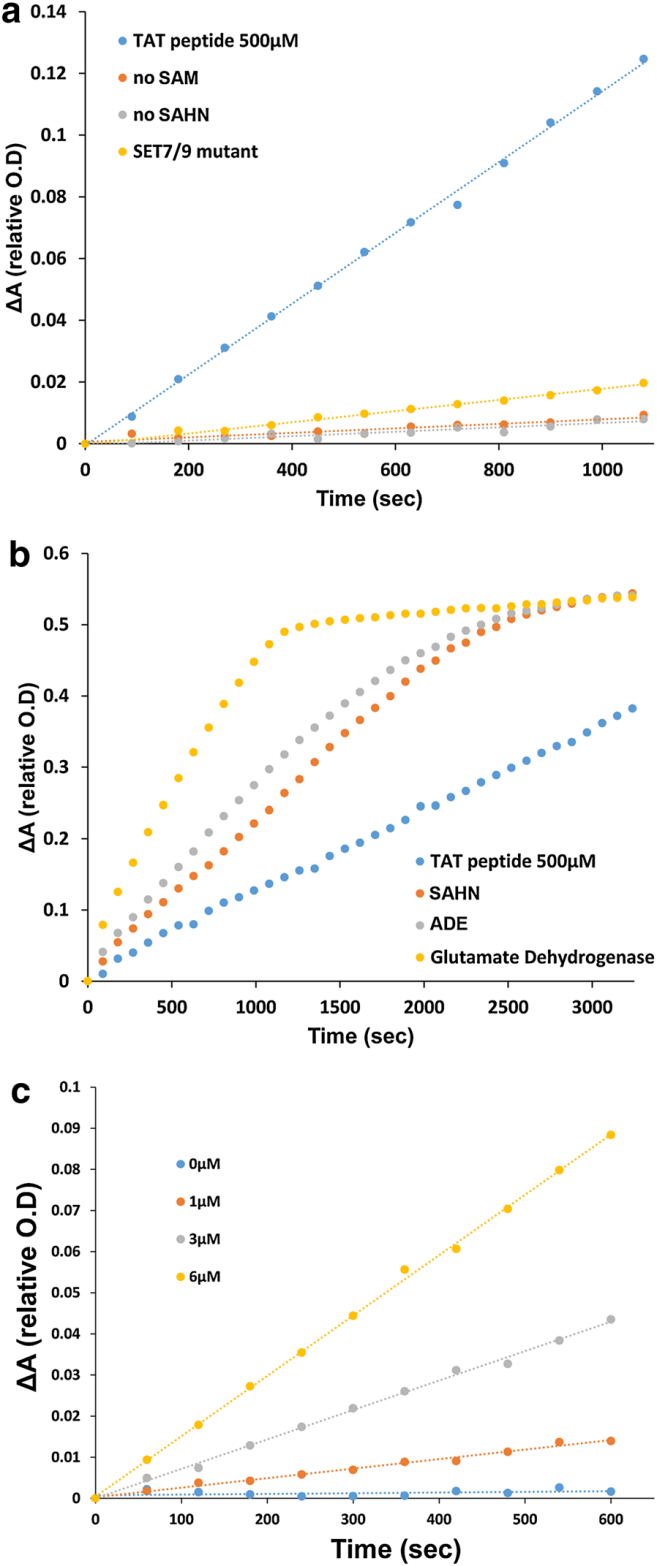
Establishment of the continuous coupled assay for MTs using short peptide as a substrate **a**. The detection of MTs activity is dependent on all assay components. The reaction cannot be monitored in the absence of SAM (*orange*), SAHN (*grey*), ADE or glutamate dehydrogenase (Additional file 1: Figure S1). In addition, very low activity was monitored in the presence of SET7/9 inactive mutant (*yellow*). Activity is measured only when all reaction components are present including the peptide substrate (TAT peptide as an example, *blue*). **b** SET7/9 activity is the rate-limiting step in the three enzyme coupled assay. Each enzyme was monitored separately to ensure the determination of SET7/9 activity. Absorbance values at 340 nm in all reactions are normalized to 1 and the change in absorbance over time is presented as 1—the absorbance value obtains for each measurement. For represented raw data of absorbance change at 340 nm please see Additional file 1: Figure S4. **c** SET7/9 activity with TAT peptide (500 μM) at increasing enzyme concentrations including 1, 3, 6 μM leads to a proportional increase in reaction rate highlighting that this is the rate-determining step for the coupled assay

To establish the continuous coupled assay for MTs activity, we initially utilized the PKMTs SET7/9 as a model enzyme. Previous works have shown that SET7/9 exhibits broad substrate specificity catalyzing methyl transfer to a variety of histone and non-histone proteins including H3, TAF10, TAT, RelA, p53 and FoxO3 [5, 32]. Methylation of these proteins by SET7/9 was shown to regulate protein–protein and protein–DNA interactions thus modulating the target protein activity [5, 33]. We initially utilized our coupled assay to monitor SET7/9 activity with a peptide substrate derived from the HIV trans-activator TAT protein. SET7/9 was previously shown to monomethylate TAT protein at K51, thus, a peptide was designed to include the K51 acceptor residue (see peptide sequence in the “Methods” section). We found that when all components of the coupled reaction are present, a gradual change in absorbance at 340 nm was observed indicating the successful coupling of SET7/9 activity with NADPH oxidation (Fig. 2a). To verify that our coupled assay specifically measures MT activity, we performed several control reactions in which one of the reaction components was omitted. We observed that when each of the coupled enzymes or SAM was absent from the reaction mix no activity was measured (Fig. 2a; Additional file 1: Figure S1). In addition, we observed that in the presence of SET7/9 variant containing the E254A mutation, very low activity was observed in accordance with previous report [34] (Fig. 2a). To further verify that SET7/9 is the rate-determining step in our kinetic measurements, we examined the catalytic rate of each step in the coupled reaction independently by monitoring NADPH oxidation. We found that under our reaction mixture (see “Methods” for details) the three coupling enzymes are significantly faster than SET7/9 activity (Fig. 2b). We have also shown that increasing in SET7/9 enzyme concentration is directly proportional to the increase in catalytic activity (Fig. 2c). To further verify that the MT reaction is the rate-determining step, we performed the reaction with doubled amount of each of the coupling enzymes (ADE, SAHN and glutamate dehydrogenase) and observed no increase in methylation rate (Additional file 1: Figure S2). Next, we examined the sensitivity of our assay by determining NADPH oxidation at limited SAH concentrations. Using this assay, we were able to detect a concentration of 170 nM of SAH highlighting that a transfer of 170 nM of methyl group to a peptide/protein substrate is detectable in our system (Additional file 1: Figure S3). Overall, these controls ensure that the change in absorbance at 340 nm reflects the true measurement of SET7/9 methylation activity and provides a wide dynamic range for activity measurements at different conditions (e.g., substrate concentrations see below).

### Measurements of SET7/9 and SETD6 activity with peptide and protein substrates

**Fig. 3:**
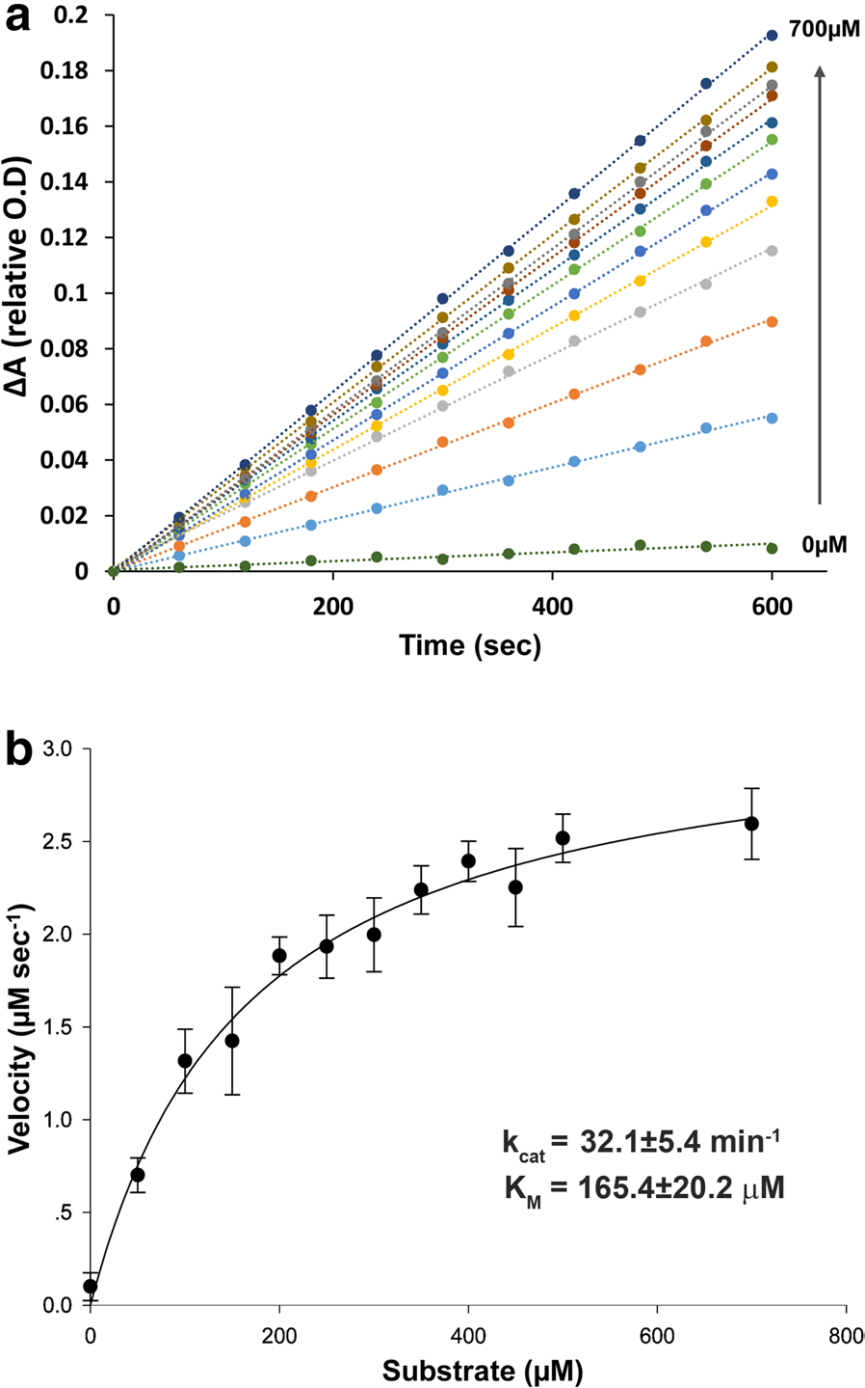
Steady-state kinetics of SET7/9 methyl transfer activity to peptide derived from FoxO3 substrate. **a** Kinetic traces of SET7/9 at increased FoxO3 peptide concentrations (0–700 µM). Initial rates at each FoxO3 substrate concentration were calculated by fitting the raw data to a linear equation. Absorbance values at 340 nm in all reactions are normalized to 1 and the change in absorbance over time is presented as 1—the absorbance value obtains for each measurement. **b** Michaelis–Menten (MM) plots for SET7/9 activity with FoxO3 peptide. The kinetic parameters derived from the fit to the MM equations are *k*_cat_ of 32.1 ± 5.4 min^−1^ and *K*_M_ of 165.4 ± 20.2 μM

To examine whether our coupled kinetic assay can be utilized for the MM kinetic analysis of SET7/9 activity, we have utilized a peptide derived from the FoxO3 protein. Previous work has shown that SET7/9 methylates FoxO3 at K271 modulating its transcriptional activity and stability [35]. Thus, we have designed a 15 amino acid peptide substrate derived from FoxO3 containing K271 to quantitatively measure SET7/9 catalytic activity (see peptide sequence in the “Methods” section). Using the coupled assay, we measured the initial rates of SET7/9 activity (5 μM) at different peptide substrate concentrations ranging from 0 to 700 μM (Fig. 3a). As expected, we observed a gradual increase in initial rates that is correlated with the increase in FoxO3 peptide concentration. Fitting the initial rate data to the MM equation allowed us to derive kinetic parameters for the activity of SET7/9 with the FoxO3 peptide (Fig. 3b). We found that the *K*_M_ and *k*_cat_ parameters for this activity are 165.4 ± 20.2 μM and 32 ± 0.023 min^−1^, respectively. The values are in excellent correlation with previous analysis of SET7/9 activity with H3K4 and DNMT1K142 utilizing radioactive MT assay [36]. The previously measured *K*_M_ and *K*_cat_ for H3K4 and DNMTK142 are 143 and 134 μM and 48 and 42 min^−1^, respectively [36]. This correlation provides strong support for our continuous assay and confirms that the FoxO3 peptide is a substrate for SET7/9 [35].

**Fig. 4:**
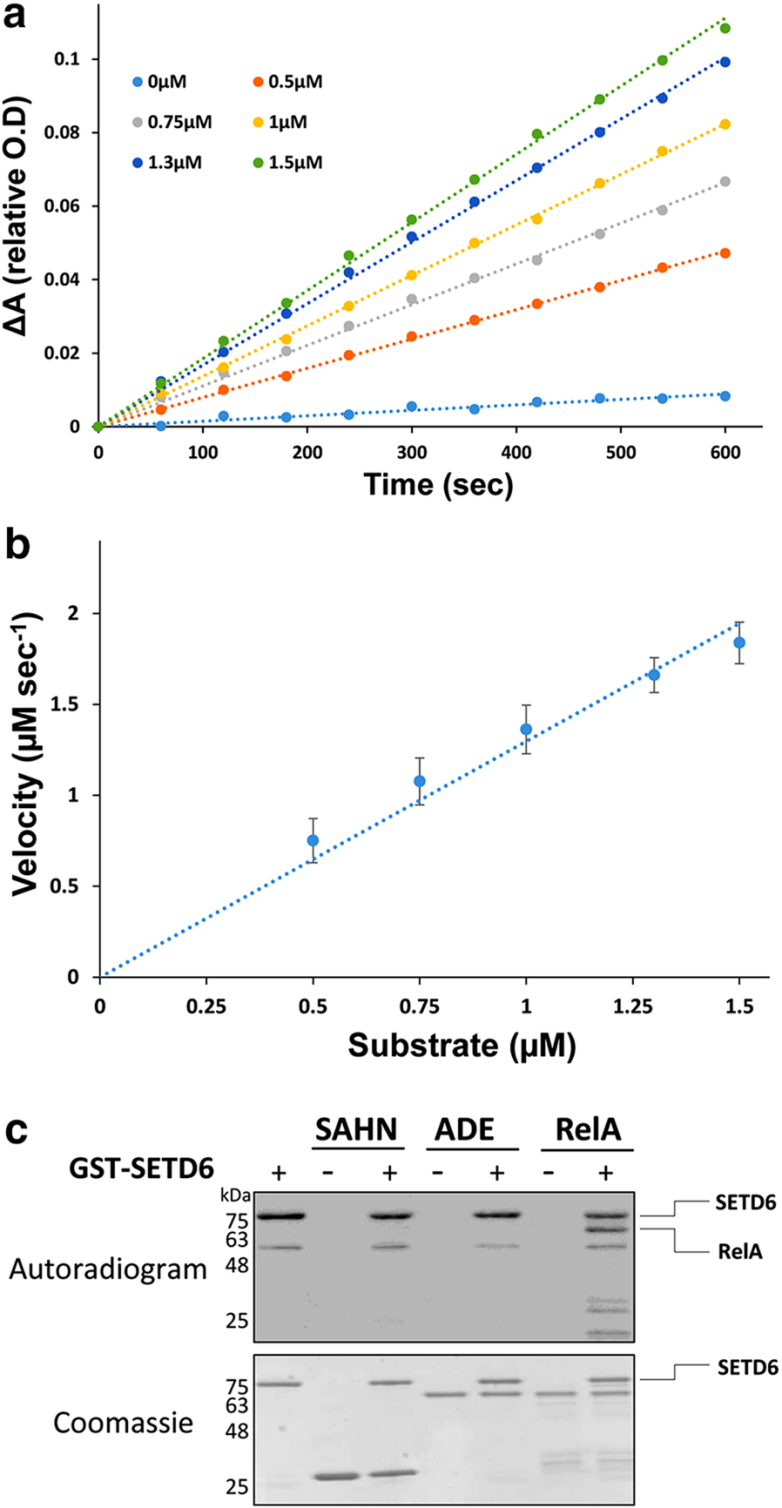
Steady-state kinetic analysis of SETD6 activity with RelA protein (residues 1–431). **a** Kinetic traces of SETD6 at increased RelA concentrations of 0–2.5 μM. Initial rate at each RelA concentration was calculated by fitting the raw data to a linear equation. Absorbance values at 340 nm in all reactions are normalized to 1 and the change in absorbance over time is presented as 1—the absorbance value obtains for each measurement. **b** Fitting the initial rate data to linear equation allows the determination of *k*_cat_/*K*_M_ to be 3.2·10^5^ s^−1^ M^−1^. Background reaction was subtracted from initial rates. **c** Radioactive gel analysis of SETD6 activity with SAHN, ADE and RelA protein (1–431) showing that methylation does not take place on SAHN and ADE and only RelA is recognized as a substrate for SETD6

To further examine whether the coupled assay can monitor MTs activity with full length protein substrates, we examined the activity of SETD6 with RelA protein (residues 1–431 [37, 38]). Previously, SETD6 was shown to methylate RelA on K310 leading to a dramatic modulation of NFκB transcriptional activity [38]. To measure SETD6 activity with RelA protein, we utilized RelA concentrations of up to 3 μM (Fig. 4a). Analysis of SETD6 activity at these RelA concentrations shows a linear increase in reaction rate with a slope of *k*_cat_/*K*_M_ of 3.2·10^5^ s^−1^ M^−1^ (Fig. 4b). These results demonstrate that our assay can be utilized to examine MTs activity with full length proteins as substrates, paving the way for additional studies to examine MTs activity with natural protein substrates. To further validate that the coupling enzymes utilized in the MTs kinetic assay do not act as substrates for methylation, we utilized radioactive [^3^H]-SAM as part of the SETD6 RelA coupled assay. The utilization of [^3^H]-SAM in the assay allows monitoring methylation of each protein in the reaction mixture using SDS-PAGE followed by autoradiography. We found that while RelA serves as a substrate for SETD6 [38], the SAH and ADE coupling enzymes do not and thus no significant background is observed in the absence of substrate (Figs. 1, 4c).

### Measurements of *M.Hae*III methyl transfer activity with DNA substrate

**Fig. 5:**
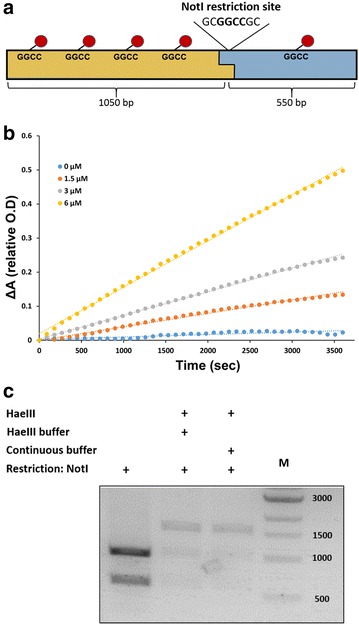
Monitoring DNA methyl transferase activity using the coupled kinetic assay. Steady-state kinetics of *M.Hae*III methyl transfer activity with DNA fragment containing six methylation sites. **a** Scheme of DNA substrate for *M.Hae*III containing six methylation sites (*red*) including one methylation site that is located at *Not*I recognition sequence. *Not*I cleavage at this site leads to two fragments of 550 and 1050 bp. **b** Kinetic traces of *M.Hae*III at increased *M.Hae*III concentrations (*blue* 1.5 μM, *orange* 3 μM and *grey* 6 μM) showing the increase in methylation rate. DNA concentration for all reactions was 400 nM. **c** DNA methylation by *M.Hae*III prevents cleavage by *Not*I. Gel electrophoresis analysis of the DNA substrate cleavage by *Not*I prior and following methylation by *M.Hae*III. Methylation of the DNA substrate by *M.Hae*III was performed in the *M.Hae*III optimal reaction buffer and in the coupled assay buffer to ensure that the later does not interfere with DNA methylation (reaction time 3 h, *M.Hae*III concentration is 2 μM)

To further examine the versatility of the assay for the general monitoring of MTs activity, we examined the methylation activity of *M.Hae*III from *Haemophilus aegyptius* with DNA substrate. *M.Hae*III belongs to a large family of bacterial DNA MTs that catalyses cytosine C5 DNA methylation [39]. *M.Hae*III methylates the internal cytosine of the canonical sequence GGCC and is utilized in the restriction-modification bacterial defense system against phage infection. To examine *M.Hae*III activity using our coupled assay, we first prepared DNA substrate by PCR amplification of 1.6 kb DNA fragment from pGex plasmid containing six predicted methylation sites for *M.Hae*III (Fig. 5a). We utilized the coupled assay to monitor *M.Hae*III activity with different DNA concentrations and measured the initial reaction rates. We observed an increase in the initial rate of *M.Hae*III catalysed DNA methylation at increased DNA substrate concentrations, demonstrating our ability to measure the kinetics of MTs with DNA substrates (Fig. 5b). To verify that *M.Hae*III methylates DNA under the coupled assay conditions, we performed *Not*I digestion analysis. Our DNA substrate contains one *Not*I cleavage site (GCGGC*CGC) that is located 1 kb from the 5′ of the DNA. Methylation of this site will prevent *Not*I cleavage leading to intact 1.6 kb substrate even in the presence of *Not*I. Indeed, we found that methylation of the DNA substrate by *M.Hae*III in the presence of all coupled assay components prevents *Not*I cleavage leading to the presence of undigested 1.6 kb fragment (Fig. 5c). These results highlight the utility of the coupled assay to efficiently monitor MTs activity with DNA substrates paving the way for quantitative analysis of many DNA methylation enzymes and sites.

## Discussion

In the past decade, MTs have attracted significant attention due to their roles in regulating central biological processes and their association with diseases [1–4]. Despite extensive investigations of the cellular roles of MTs, much less is known about their biochemical functions, substrate recognition and catalytic mechanism. The establishment of biochemical assay to readily monitor MTs activity will greatly facilitate such mechanistic research. Previously, several groups have attempted to establish biochemical assays for monitoring MTs activity; however, each approach suffers for different draw backs (see “Background” section).

Our assay is based on monitoring SAH formation by three-enzyme coupled reaction leading to the formation of ammonia and the oxidation of NADPH. Utilization of the different enzymes raises the concern that the enzyme used for the coupled reactions can serve as substrates for MTs. Utilizing a radioactive assay, we have shown that SETD6 does not methylate any of the coupling enzymes (Fig. 4c). Previously, the coupling of ammonia formation with α-ketoglutarate activity and NADPH oxidation was utilized for the establishment of Sirt1 deacetylase activity [40, 41]. In addition, the coupling of enzymatic transfer reactions to NADPH oxidation was extensively utilized for the detection of a wide range of glycosyltransferases for the quantitative determination of their catalytic activities [42]. To monitor glycosyltransferase activity, dinucleotides (e.g., UDP, or GDP) generated following sugar transfer activity are coupled to NADH oxidation by pyruvate kinase and lactate dehydrogenase [42, 43]. Thus, coupling different enzymatic reactions to glutamate dehydrogenase activity, enabling monitoring these reactions at 340 nm, is a common tool for enzymatic activity determination.

Our coupled assay allows the continuous monitoring of MTs activity (Fig. 1). We believe that this assay can be used as a fast, convenient and inexpensive approach for the detailed biochemical characterization of a wide variety of MTs. Since the assay can be performed in a multi-well plate format, it can be readily adapted for high-throughput screening of MTs activity. Such screening can be highly useful in the search for new MTs inhibitors or activators. Since the coupling of three enzymes is required to monitor MTs activity, appropriate controls must be taken to ensure the specificity of the new inhibitors to the target methyltransferase.

## Conclusion

In conclusion, we have developed a rapid, continuous three-enzyme coupled UV absorption assay for the characterization of enzymes that use SAM to catalyze methyl transfer reactions. We have shown the versatility and robustness of this assay in monitoring the activity of two different protein methyl transferases SET7/9 and SETD6 utilizing peptide and protein substrates, respectively. In addition, we showed that the coupled assay can be utilized for monitoring DNA methylation kinetics using *M.Hae*III as a model enzyme.

## Methods

### Molecular biology

*SET7/9* gene was cloned into pET-Duet plasmid for *E. coli* expression and purification. The *SET7/9* gene was PCR amplified from pGex-6p1 plasmid containing the gene as a template. The amplified DNA fragment was then cleaved by *Spe*I and *Xho*I restriction enzymes and ligated into a pET-Duet plasmid containing a maltose binding domain (MBP) tag. The adenine deaminase (ADE, E.C. 3.5.4.4) and *S*-adenosyl-l-homocysteine nucleosidase (SAHN, E.C. 3.2.2.9) genes were amplified from the genomic DNA of an XL1 blue *E. Coli* strain using PCR. The amplified DNA fragments were then cleaved using *Nhe*I and *Hind*III restriction enzymes and ligated into a pET28a plasmid containing a Hisx6 tag at the N-terminal of the protein.

### Protein expression and purification

SET7/9 enzyme was expressed from a pET-Duet plasmid containing the *SET7/9* gene fused to a MBP tag in *E. Coli* BL21 (DE3). Expression was induced using 0.5 mM isopropyl b-d-1-thiogalactopyranoside (IPTG) for 6 h at 30 °C. Following inductions, cells were centrifuged and resuspended at a buffer containing 25 mM Tris–HCL (pH 7.5), 200 mM NaCl and 1 mM DTT. The cells were lysed using a French press and the resulting cell extract was centrifuged at 13,000*g* for 1 h. The SET7/9 protein was then purified from the clear lysate using amylose beads (Amersham) according to standard procedures. The MBP tag was then cleaved using TEV protease for 4 h at 4 °C and purified using gel-filtration chromatography to obtain a monomeric SET7/9 protein. GST-SETD6 was expressed and purified as previously described [38].

The RelA protein (residues 1–431) was expressed using a pGEX-6p1 plasmid containing the *RelA* gene fused to a GST tag in *E. Coli* BL21 (DE3). Expression was induced with 0.5 mM IPTG for 16 h at 20 °C. Following inductions, cells were centrifuged and resuspended in buffer containing 25 mM Tris–HCL (pH 7.5), 200 mM NaCl and 1 mM DTT. The cells were lysed using a French press and the cell extract was centrifuged at 13,000*g* for 1 h. The RelA protein was then purified using glutathione beads (Amersham) according to standard procedures.

The ADE enzyme was expressed from pET28a plasmid containing the ADE gene fused to a 6xHis tag at the N-terminal in *E.coli* BL21 (DE3). Expression was induced with 0.5 mM IPTG for 16 h at 20 °C. To replace the Fe^2+^ metal at the ADE active site with Mn^2+^, 50 μM of 2,2′-dipyridyl and 1.0 mM MnCl_2_ were added at time of the induction [44]. Following induction, cells were centrifuged and resuspended at a buffer containing 25 mM Tris–HCL (pH 7.5), 200 mM NaCl and 1 mM DTT. The cells were lysed using French press and the cell extract was centrifuged at 13,000*g* for 1 h. The ADE protein was then purified using nickel-NTA beads using standard procedures. Following purification, a dialysis against the original buffer was performed to remove the imidazole. The SAHN protein was purified using the same purification protocol without the metal replacement procedure performed for the ADE purification. For DNA methylation assays, commercial *M.Hae*III enzyme was purchased from NEB.

### Methylation assays

The continuous coupled methylation assay was carried out with clear flat bottom 96 well plates, containing 4.5 µM SAHN, 3 µM ADE, 2.62 units of glutamate dehydrogenase (Ammonia detection kit, Sigma), 300 µM SAM and a varying concentration of methyltransferase enzyme and methyl acceptor. A concentration of 300 µM SAM was used to ensure saturation of the methyl donor. A final volume of 250 µl was reached in the well using the ammonia assay kit buffer (Sigma). The assay was performed at 30 °C, and the reaction was monitored at 340 nm using Tecan Infinite M200 plate reader. Kinetic parameters were derived by fitting to Michaelis–Menten V_o_ = *k*_cat_[E]_0_[S]_0_/([S]_0_ + *K*_M_) model. The TAT and FoxO3 peptide substrate sequences are GISYGRK**K**RRQRRRP (residues 44–58) and RGRAAKK**K**AALQTA (residues 264–277), respectively (methylated lysine is in bold). For deriving kinetic parameters, three replicates of each substrate concentration were used. All reactions were performed at 30 °C and were measured for a period of 10 min up to 1 h (Figs. 2, 3, 4, 5). The buffer for all enzymes is 25 mM Tris pH 7.5, 200 mM NaCl and 1 mM DTT supplemented with the buffer from the ammonia detection kit for the glutamate dehydrogenase activity (Sigma). As a substitution for the ammonia detection kit, we used a defined reaction conditions including Tris 25 mM pH7.5, bovine serum albumin (BSA) 0.5 % (v/v), 300 mM SAM, α-ketoglutarate 5 mM, NADPH 0.5 mM, SAHN 5 µM, ADE 3.5 µM and 2.62 units of glutamate dehydrogenase (Additional file 1: Figure S5). The rate of methyl transfer was still higher utilizing the Sigma buffer for ammonia detection (Ammonia detection kit, Sigma).

### Radioactive in vitro methylation assay

Assays were performed as previously described [38]. Briefly, recombinant proteins were incubated with recombinant SETD6, and 2 mCi ^3^H-SAM (Amersham Pharmacia Biotech Inc, Piscataway, NJ, USA) in methylation buffer [50 mM Tris–HCL (pH 8.0), 10 % glycerol, 20 mM KCl, 5 mM MgCl_2_ and 1 mM PMSF] at 30 °C overnight. The reaction mixture was resolved by SDS-PAGE, followed by either autoradiography or Coomassie blue stain.

## Additional file

10.1186/s13072-015-0048-y Establishment of the continuous coupled assay for MTs using short peptide as a substrate **a**. The detection of MTs activity is dependent on all assay components. The reaction cannot be monitored in the absence of glutamate dehydrogenase (orange) or adenine deaminase (ADE, grey). Activity is measured only when all reaction components are present including the peptide substrate (TAT peptide as an example, blue). **Figure S2.** Methyltransferase activity is the rate-determining step of NADPH oxidation. Doubling the concentration of each component in the total methyltransferase reaction does not lead to increase in methylation rate (**A**) SET7/9 (5 μM) activity with TAT peptide (500 μM) at 3 μM (blue) or 6 μM (orange) of adenine deaminase (ADE), 2.62 units (blue) or 5.24 units (orange) of glutamate dehydrogenase and 4.5 μM (blue) or 9 μM (orange) of SAH nucleosidase. (**B**) ADE activity with 3 μM (blue) or 6 μM (orange) of the enzyme at 150 μM concentration of adenine (**C**) Glutamate dehydrogenase activity with 2.62 units (blue) or 5.24 units (orange) of the enzyme at 30 μM NH_4_^+^ concentration. (**D**) SAH nucleosidase activity 4.5 μM (blue) or 9 μM (orange) with 100 μM SAH concentration. **Figure S3.** Monitoring of SAHN activity at limiting SAH (Sigma) concentrations using the coupled assay. Activity was detected using SAHN 5 µM, ADE 3.5 µM and 2.62 units of glutamate dehydrogenase in the presence of 300 µM SAM by monitoring changes at 340 nm. **Figure S4.** Raw measurement of MT activity coupled with NADPH oxidation. A. The rate of absorbance decrease at 340 nm reflects the rate of SET7/9 activity with the TAT peptide (see also **Figure S1** and Figure 2 main paper). **B**. Absolute absorbance values are transformed to change in absorbance at 340 nm and the values of 1-absorbance change are shown to highlight the rate of methylation. **Figure S5.** SET7/9 activity with different FoxO3 peptide concentrations in a defined reaction conditions including Tris 25 mM pH 7.5, bovine serum albumin (BSA) 0.5 % (v/v), 300 mM SAM, α-ketoglutarate 5 mM, NADPH 0.5 mM, SAHN 5 µM, ADE 3.5 µM and 2.62 units of glutamate dehydrogenase. Activity increases at increased peptide concentration.

## Abbreviations

MT: methyltransferase
SAM: *S*-adenosylmethionine
SAH: *S*-adenosylhomocysteine
ADE: adenine deaminase
GST: glutathione *S*-transferase
MBP: maltose binding domain
