# Supplementary material for: A continuous kinetic assay for protein and DNA methyltransferase enzymatic activities
Source: Epigenetics Chromatin. 2015 Dec 15;8:56. doi: 10.1186/s13072-015-0048-y (PMC4678762; doi:10.1186/s13072-015-0048-y)
Supplement: Supplementary file 1 — 10.1186/s13072-015-0048-y Establishment of the continuous coupled assay for MTs using short peptide as a substrate a. The detection of MTs activity is dependent on all assay components. The reaction cannot be monitored in the absence of glutamate dehydrogenase (orange) or adenine deaminase (ADE, grey). Activity is measured only when all reaction components are present including the peptide substrate (TAT peptide as an example, blue). Figure S2. Methyltransferase activity is the rate-determining step of NADPH oxidation. Doubling the concentration of each component in the total methyltransferase reaction does not lead to increase in methylation rate (A) SET7/9 (5 μM) activity with TAT peptide (500 μM) at 3 μM (blue) or 6 μM (orange) of adenine deaminase (ADE), 2.62 units (blue) or 5.24 units (orange) of glutamate dehydrogenase and 4.5 μM (blue) or 9 μM (orange) of SAH nucleosidase. (B) ADE activity with 3 μM (blue) or 6 μM (orange) of the enzyme at 150 μM concentration of adenine (C) Glutamate dehydrogenase activity with 2.62 units (blue) or 5.24 units (orange) of the enzyme at 30 μM NH4 + concentration. (D) SAH nucleosidase activity 4.5 μM (blue) or 9 μM (orange) with 100 μM SAH concentration. Figure S3. Monitoring of SAHN activity at limiting SAH (Sigma) concentrations using the coupled assay. Activity was detected using SAHN 5 µM, ADE 3.5 µM and 2.62 units of glutamate dehydrogenase in the presence of 300 µM SAM by monitoring changes at 340 nm. Figure S4. Raw measurement of MT activity coupled with NADPH oxidation. A. The rate of absorbance decrease at 340 nm reflects the rate of SET7/9 activity with the TAT peptide (see also Figure S1 and Figure 2 main paper). B. Absolute absorbance values are transformed to change in absorbance at 340 nm and the values of 1-absorbance change are shown to highlight the rate of methylation. Figure S5. SET7/9 activity with different FoxO3 peptide concentrations in a defined reaction conditions including Tris 25 mM pH 7.5, bovine ser [file 13072_2015_48_MOESM1_ESM.docx]

**Supplementary Information**

**A continuous kinetic assay for protein and DNA methyltransferase enzymatic activities**

Shai Duchin^1,3^, Zlata Vershinin^2,3^, Dan Levy^2,3*^ and Amir Aharoni^1,3*^

^1^Departments of Life Sciences and ^2^Microbiology, Immunology and Genetics, ^3^The National Institute for Biotechnology in the Negev (NIBN), Ben-Gurion University of the Negev, Be’er Sheva 84105, Israel.

*Equal Contribution and Correspondence

Correspondence should be addressed to A.A. ([aaharoni@bgu.ac.il](mailto:aaharoni@bgu.ac.il)) or D.L (ledan@post.bgu.ac.il)

Email addresses:

Shai Duchin - shaiduchin@gmail.com Dan Levy- ledan@post.bgu.ac.il

Zlata Vershinin- [zlatave@post.bgu.ac.il](mailto:zlatave@post.bgu.ac.il) Amir Aharoni- [aaharoni@bgu.ac.il](mailto:aaharoni@bgu.ac.il)


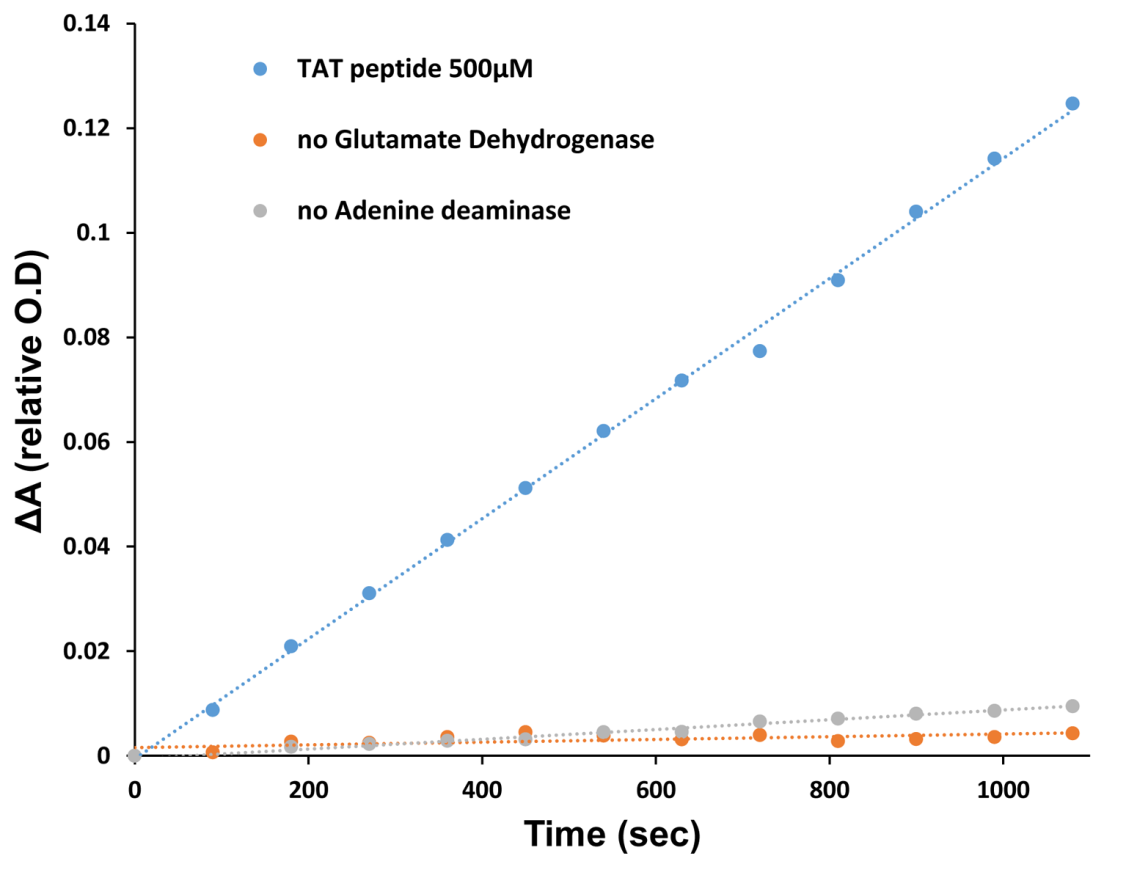


**Figure S1**: Establishment of the continuous coupled assay for MTs using short peptide as a substrate **a**. The detection of MTs activity is dependent on all assay components. The reaction cannot be monitored in the absence of Glutamate dehydrogenase (orange) or adenine deaminase (ADE, grey). Activity is measured only when all reaction components are present including the peptide substrate (TAT peptide as an example, blue).


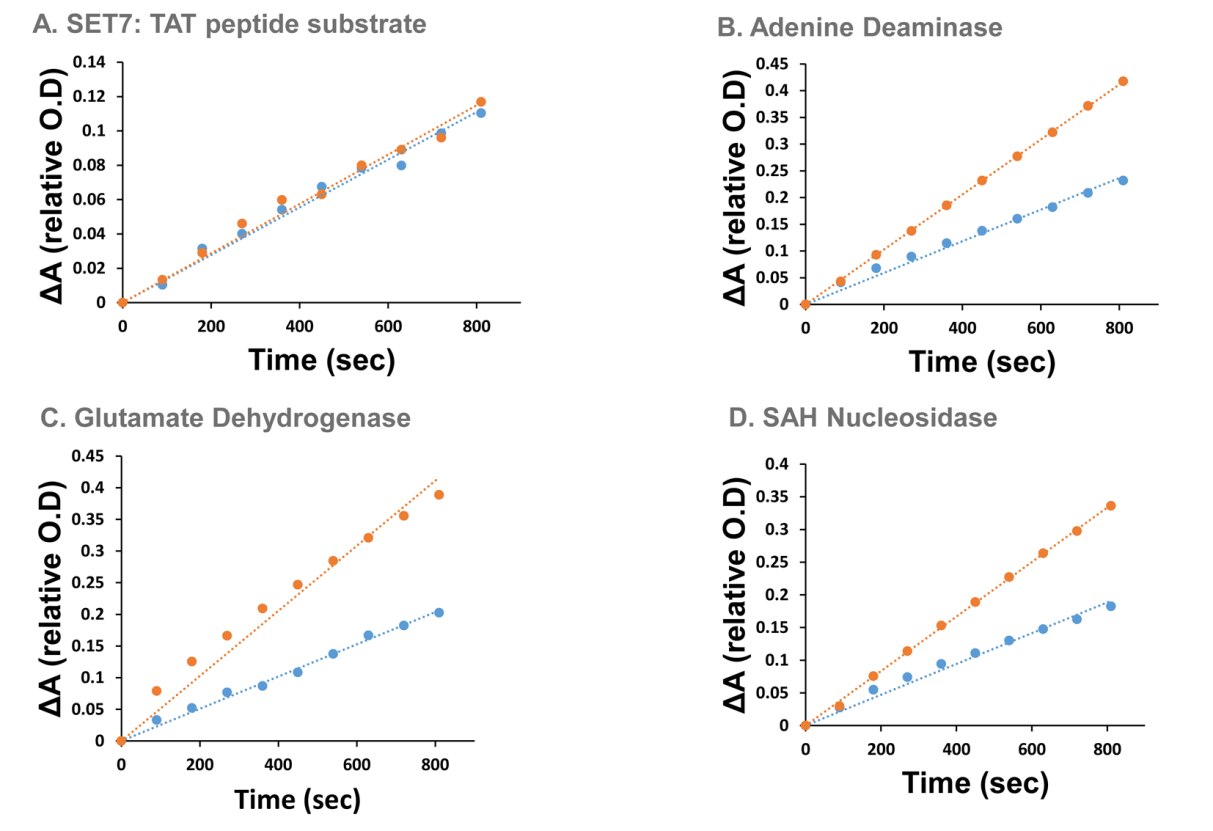


**Figure S2:** Methyltransferase activity is the rate determining step of NADPH oxidation. Doubling the concentration of each component in the total methyltransferase reaction does not lead to increase in methylation rate (**A**) SET7/9 (5 μM) activity with TAT peptide (500 μM) at 3 μM (blue) or 6 μM (orange) of Adenine deaminase (ADE), 2.62 units (blue) or 5.24 units (orange) of Glutamate dehydrogenase and 4.5 μM (blue) or 9 μM (orange) of SAH nucleosidase. (**B**) ADE activity with 3 μM (blue) or 6 μM (orange) of the enzyme at 150 μM concentration of adenine (**C**) Glutamate dehydrogenase activity with 2.62 units (blue) or 5.24 units (orange) of the enzyme at 30 μM NH_4_^+^ concentration. (D) SAH nucleosidase activity 4.5 μM (blue) or 9 μM (orange) with 100 μM SAH concentration.


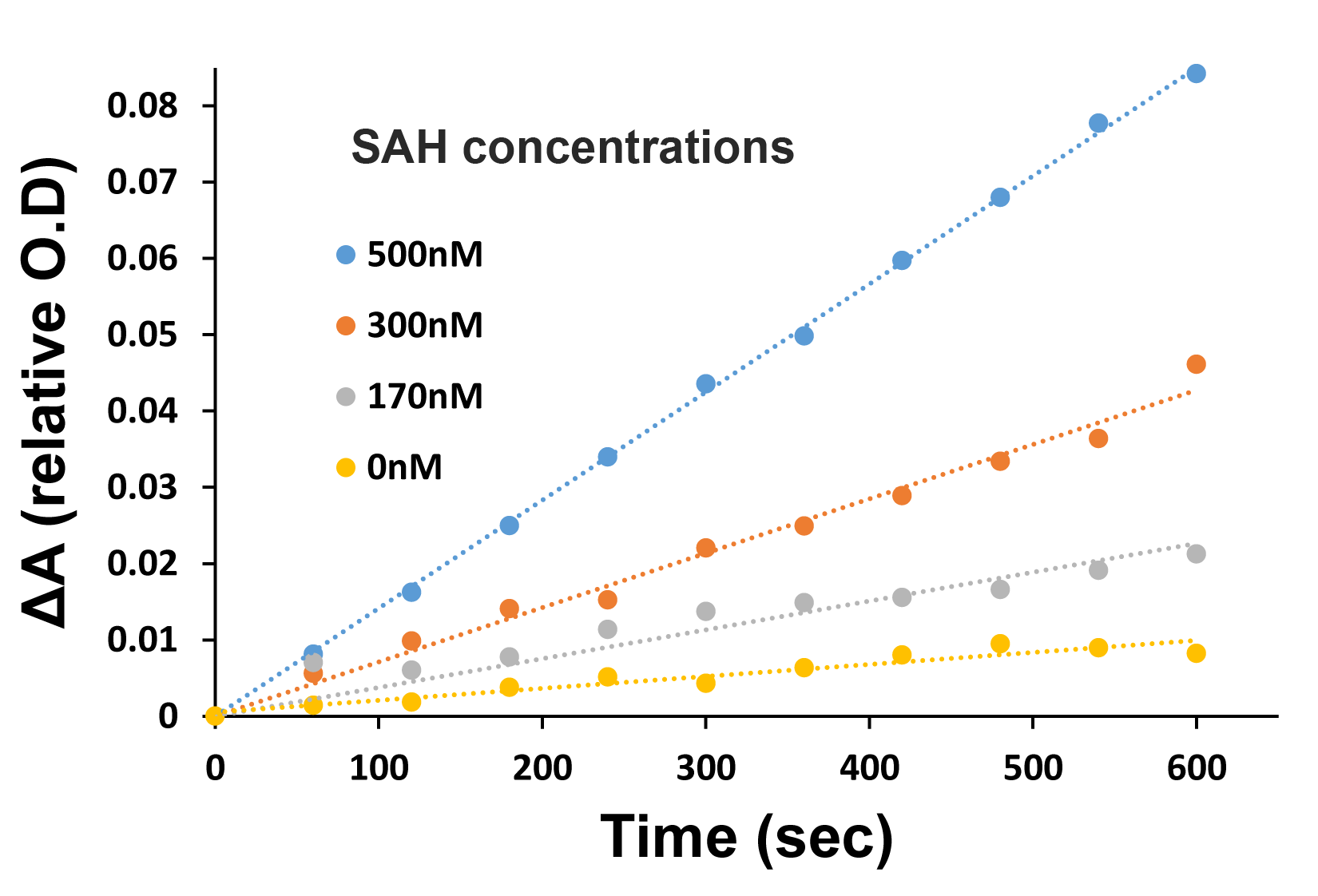


**Figure S3**: Monitoring of SAHN activity at limiting SAH (Sigma) concentrations using the coupled assay. Activity was detected using SAHN 5µM, ADE 3.5 µM and 2.62 Units of Glutamate dehydrogenase in the presence of 300 µM SAM by monitoring changes at 340 nm.


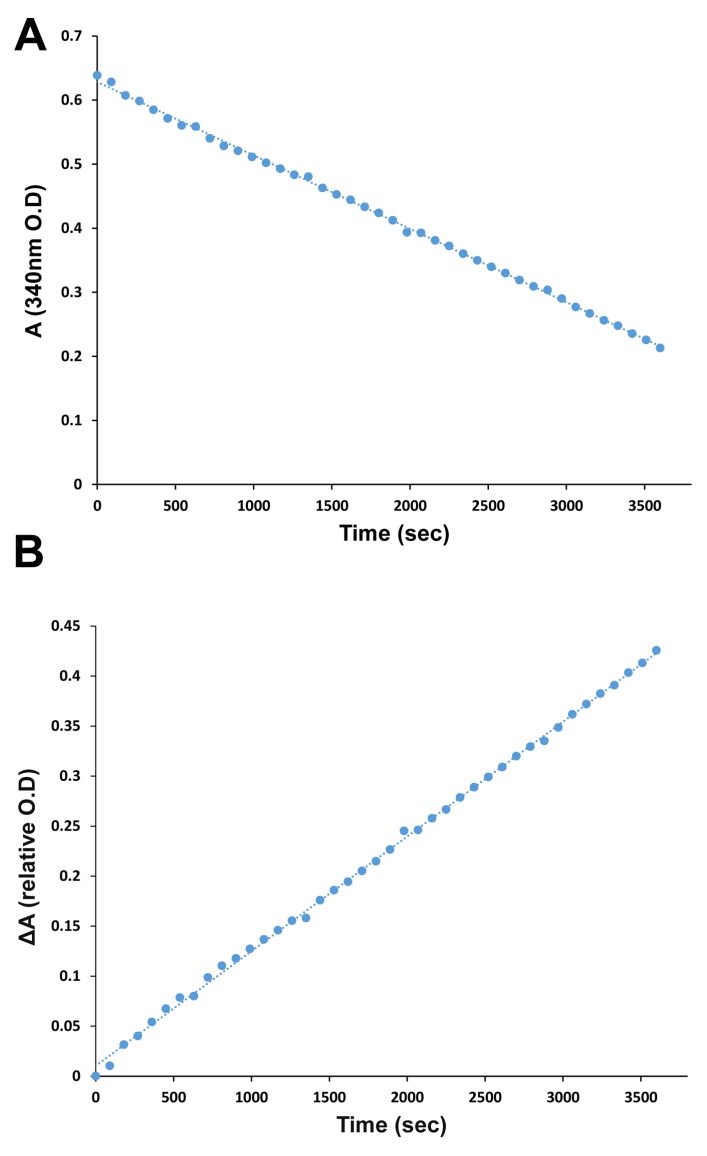


**Figure S4**: Raw measurement of MT activity coupled with NADPH oxidation. A. The rate of absorbance decrease at 340 nm reflects the rate of SET7/9 activity with the TAT peptide (see also **Figure S1** and **Figure 2** main paper). **B**. Absolute absorbance values are transformed to change in absorbance at 340 nm and the values of 1-absorbance change are shown to highlight the rate of methylation.


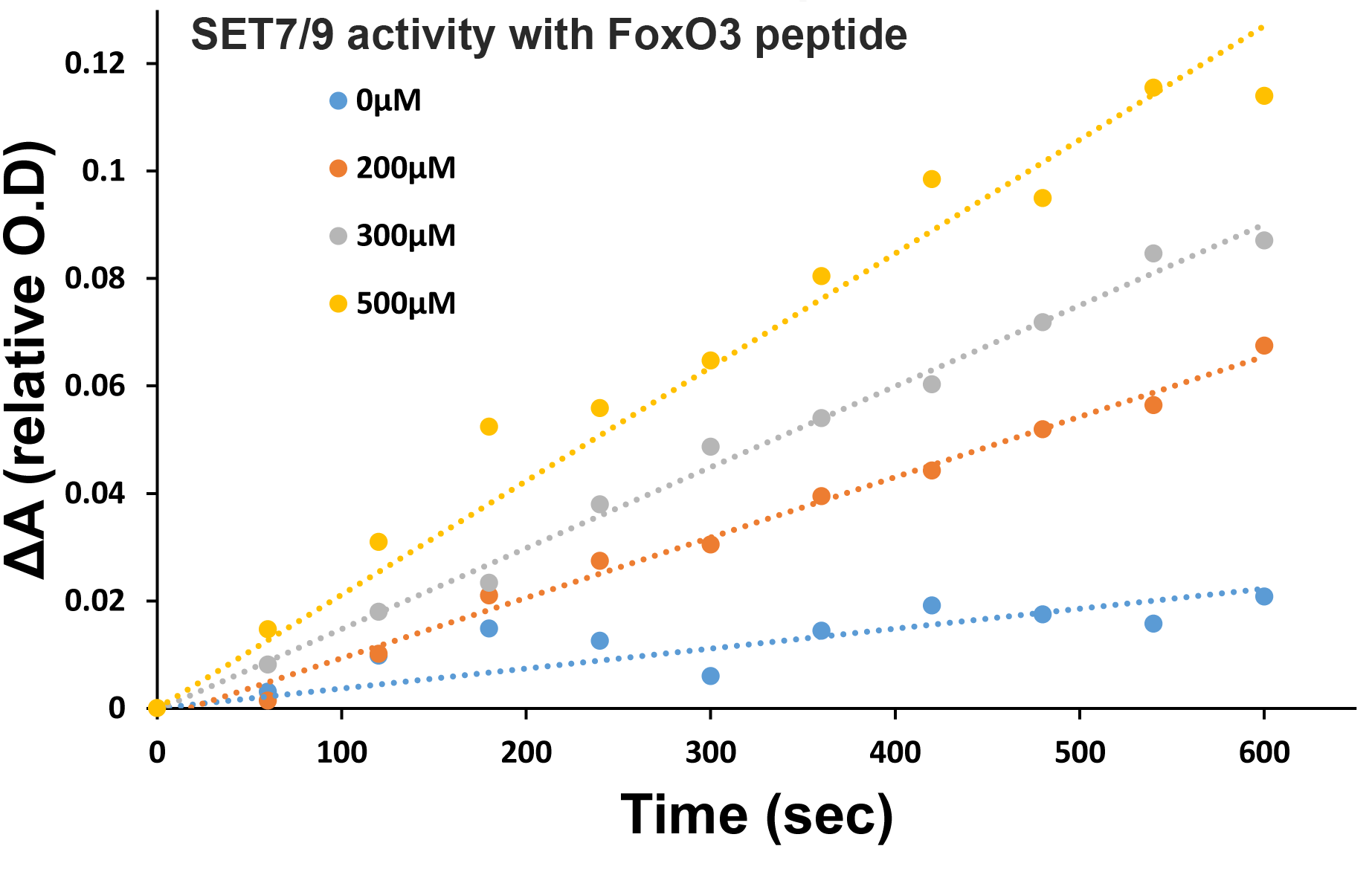


**Figure S5**: SET7/9 activity with different FoxO3 peptide concentrations in a defined reaction conditions including Tris 25mM pH 7.5, bovine serum albumin (BSA) 0.5% (v/v), 300 mM SAM, a-ketoglutarate 5 mM, NADPH 0.5 mM, SAHN 5µM, ADE 3.5 µM and 2.62 Units of Glutamate dehydrogenase. Activity increases at increased peptide concentration.
